# The patient path to diagnosis of atrial fibrillation: a qualitative study in primary care

**DOI:** 10.3399/BJGPO.2025.0006

**Published:** 2025-09-10

**Authors:** Patricia N Apenteng, Veronica Nanton, Trudie Lobban, Richard Lilford

**Affiliations:** 1 Department of Applied Health Sciences, University of Birmingham, Birmingham, UK; 2 Warwick Medical School, University of Warwick, Coventry, UK; 3 Atrial Fibrillation Association, Stratford-upon-Avon, UK

**Keywords:** atrial fibrillation, detection, primary care

## Abstract

**Background:**

Atrial fibrillation (AF) is underdiagnosed and approximately 10% of ischaemic strokes occur in people with unrecognised AF.

**Aim:**

To explore the patient path to diagnosis of AF and identify ways to improve detection.

**Design & setting:**

Qualitative study in UK primary care.

**Method:**

We interviewed patients with a recent diagnosis of AF (<6 months) to understand their path to diagnosis, and interviewed primary care clinicians to explore their experience of detecting AF. The data were analysed using framework analysis.

**Results:**

Thirty patients and ten primary care clinicians were interviewed. Patients with non-specific symptoms generally did not perceive the symptoms as serious, and many delayed seeing a healthcare professional. Their experiences in primary care aligned with findings from interviews with primary care clinicians, who acknowledged that AF may not necessarily be the initial suspicion when a patient presents with certain non-specific symptoms. Primary care clinicians described narratives of good practice in the form of opportunistic pulse palpation, and challenges of detecting AF in primary care such as lack of access to Holter tests and limited opportunities to detect AF as a result of remote consultations and healthcare assistants taking on more responsibilities.

**Conclusion:**

Our findings suggest that increased public awareness of AF could improve symptom appraisal and help-seeking from healthcare professionals. Recommending opportunistic pulse palpation in primary care is also indicated. Access to Holter tests and other devices in primary care may help reduce delays in diagnosis.

## How this fits in

Atrial fibrillation (AF) is underdiagnosed, leaving patients at risk of AF-related stroke. Our study explored the patient path to AF diagnosis and identified patient and health system factors that delay diagnosis. Older patients did not recognise the significance of non-specific symptoms and delayed consulting a healthcare professional. Disparities in the capability and capacity to detect AF in primary care led to further diagnostic delays for some patients.

## Introduction

Atrial fibrillation (AF) is the most common clinically significant arrhythmia in the adult population worldwide.^1^ The prevalence of AF increases with age; in the UK prevalence increases from 7.2% in those aged ≥65 years to 10.3% in those aged ≥75 years.^2^ Patients with AF have a five-fold increase in risk of stroke and double the risk of death compared with patients without AF.^3^ Anticoagulation therapy dramatically reduces the risk of AF-related stroke and death, with a 68% relative risk reduction for stroke and 25% reduction in mortality.^4,5^ However, AF is underdiagnosed,^6,7^ and 10% of ischaemic strokes are associated with unrecognised AF.^8^

The current National Institute for Health and Care Excellence (NICE) guidelines recommend a manual pulse palpation to assess the presence of an irregular pulse in patients with breathlessness, palpitations, syncope, dizziness, chest discomfort, or a history of stroke or transient ischaemic attack.^9^ The guidelines recommend an electrocardiogram (ECG) to make a diagnosis of AF if an irregular pulse is detected.^9^ Nevertheless, detection and diagnosis of AF can be challenging in practice because AF is frequently intermittent and an irregular heart rhythm may not be present when an ECG is performed.

The UK National Screening Committee does not currently recommend screening for AF;^10^ however, a large randomised trial is underway to evaluate routine screening for AF.^11^ In the absence of a screening programme, an understanding of the patient pathways to diagnosis may provide insights to facilitate detection. This study aimed to explore the patient journey to a diagnosis of AF through interviews with patients and healthcare professionals.

## Method

### Study design

We conducted a qualitative interview study with patients with a recent diagnosis of AF (<6 months) and with primary care clinicians. We developed topic guides to explore patient factors relating to the detection of AF and identify any related clinician and systemic factors (see Supplementary Boxes S1 and S2). We applied Andersen’s model of total patient delay as a guide to understand the trajectory to diagnosis.^12^ Andersen’s model breaks the patient journey to diagnosis into three intervals: 1) the appraisal interval — the period between detecting a symptom and inferring illness; 2) the help-seeking interval — the period between deciding an illness requires medical attention and the first consultation with a healthcare professional; and 3) the diagnosis interval — the period between first consultation with a healthcare professional and diagnosis.^12^

The study was approved by an NHS Research Ethics Committee and all participants gave written consent before interviews.

### Patient and public involvement

The study was developed with the input of patients and in partnership with the AF Association, a UK-registered charity that focuses on raising awareness of AF. Two patients with AF supported the study as patient and public involvement (PPI) contributors, and were involved in the development of interview topic guides and the interpretation of study findings.

### Recruitment

We recruited patients with a recent diagnosis of AF and primary care clinciians for one-to-one interviews. The eligibility criteria for patients were: men and women aged ≥50 years, with a diagnosis of AF within the last 6 months, and the ability to provide informed consent. Twelve GP practices were purposively recruited from all six counties in the West Midlands, with the support of the West Midlands Clinical Research Network (CRN). We aimed for diversity in terms of list size, ethnic mix of the locality, and rural/urban classification (Table 1). The CRN nurses searched for patients meeting eligibility criteria in the participating practices and sent out letters inviting them to participate. The CRN also sent invitations to practice teams in the West Midlands to participate in clinician interviews. Patients and clinicians who were interested in participating then contacted the research team.

**Table 1.** Profile of participating practices, *N* = 12

Characteristic	Practices, *n* (%)
**West Midland County**	
West Midlands	5 (42.0)
Shropshire	1 (8.3)
Warwickshire	2 (16.7)
Worcestershire	1 (8.3)
Herefordshire	1 (8.3)
Staffordshire	2 (16.7)
**List size**	
<6000	2 (16.7)
6000–10 000	6 (50.0)
>10 000–30 000	1 (8.3)
>30 000	3 (25.0)
**Geography**	
Urban	10 (83.3)
Rural	2 (16.7)
**Town-level minority ethnic population, %**	
<5	2 (16.7)
5–20	5 (42.0)
21–50	2 (16.7)
>50	3 (25.0)

### Data collection

Interviews with patients and primary care clinicians were conducted between January and August 2023. Interviews with patients were conducted face-to-face and within 6 months of diagnosis. A narrative approach was used for the patient interviews, using a topic guide starting with an open-ended question that gave patients the freedom to tell their story in a way that was meaningful to them.^13^ The researcher followed up with questions to fill in any gaps in the story.

Interviews with healthcare professionals were conducted via Zoom. All participants signed a consent form before being interviewed. The interviews were audio-recorded and transcribed, except one interview with a stroke survivor who had dysarthria, during which the interviewer took extensive notes instead. The interviewer (PNA) was a professional researcher (PhD) with proficiency in qualitative research.

### Data analysis

The interviews were professionally transcribed. Two researchers independently reviewed three patient transcripts and three clinician transcripts and developed codes. The coding framework was developed iteratively with input from the team and PPI contributors, and applied to the remaining transcripts. The data were then analysed using framework analysis^14^ and interpreted through discussion between the study team. Supplementary Box S3 and S4 provide excerpts of the data analysis.

## Results

### Participants

Thirty patients were interviewed. Supplementary Table S1 shows the number of patients invited, the number of responses received, and the number of patients interviewed per practice.

Of the participating patients, 43% were female and 60% were aged ≥75 years (Table 2). Participants were mostly of white ethnicity (*n* = 29/30) and 77% were retired while the remainder worked part-time. Participants had a mix of educational attainment, with secondary school being the highest level of education for 23%, and 37% having a degree or higher degree. The majority of patients (80%) experienced symptoms before diagnosis. Most had comorbidities; four had a history of stroke, with two having had a stroke at least 1 year before diagnosis and two being diagnosed with AF in a stroke ward.

**Table 2.** Patient characteristics (*N* = 30)

Variable	Patients, *n* (%)
**Sex, male**	17 (57)
**Age group, years**	
60–64	4 (13)
65–74	8 (27)
75–84	14 (47)
≥85	4 (13)
**Ethnicity**	
White	29 (97)
Mixed	1 (3)
**Highest level of education**	
Secondary school	7 (23)
College/apprenticeship	6 (20)
Professional qualification	6 (20)
Degree and above	11 (37)
**Employment status**	
Retired	23 (77)
Working part-time	7 (23)
**Employment type**	
Operational/technical	12 (40)
Professional	14 (47)
Supervisor/manager	4 (13)
**Experienced symptoms before diagnosis**	26 (87)

Ten primary care clinicians responded to the invitation and all, comprising nine GPs and one practice nurse, were interviewed (Table 3). They had been in general practice for between 4 and 14 years, and 60% were female.

**Table 3.** Characteristics of primary healthcare professionals

Study ID	Role	Sex	Years in general practice	Clinical interest	List size	Area (county)	Urban/rural
HCP01	GP	Female	5	Frailty	>30 000	West Midlands	Urban
HCP02	GP	Male	11	Cardiovascular disease	<6000	West Midlands	Urban
HCP03	GP	Female	9	Cardiology	6000–10 000	Herefordshire	Rural
HCP04	GP	Male	4	Mental health	6000–10 000	West Midlands	Urban
HCP05	Nurse	Female	5	None	>10 000–30 000	West Midlands	Urban
HCP06	GP	Male	14	Neurology/cardiology	>10 000–30 000	Staffordshire	Urban
HCP07	GP	Male	4	Frailty/older adults	6000–10 000	West Midlands	Urban
HCP08	GP	Female	12	Women’s health	>10 000–30 000	West Midlands	Urban
HCP09	GP	Female	9	Palliative care	>10 000–30 000	Shropshire	Urban
HCP10	GP	Female	5	Palliative care	>10 000–30 000	West Midlands	Urban

### Findings from interviews

**Figure 1.**
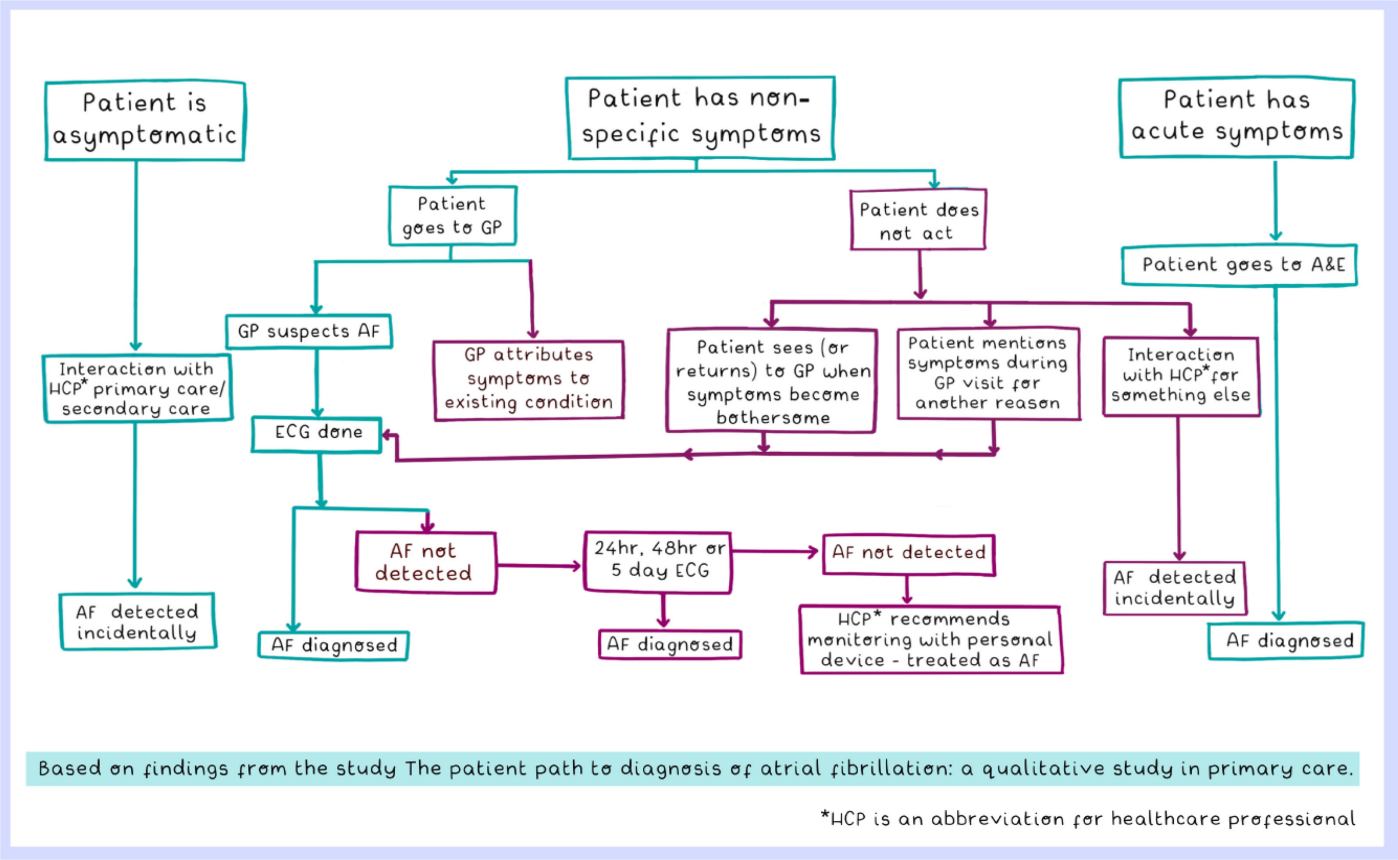
Pathways to AF diagnosis crafted from patient interviews. A&E = accident and emergency. AF = atrial fibrillation. ECG = electrocardiogram.

The pathways to diagnosis of the patients in the study are demonstrated in Figure 1. The factors that influence the patient pathways to diagnosis are described in six themes: symptom appraisal; lack of awareness; patients’ experiences in primary care; clinician knowledge and practice; digital technologies; and constraints on AF detection in primary care.

#### Symptom appraisal

Most participants experienced symptoms before diagnosis. Participants described symptoms such as breathlessness, reduced exercise tolerance, dizziness, fatigue, and palpitations, and they varied in severity. The range of symptoms described by patients is presented in Supplementary Box S5. Patients with acute onset of symptoms perceived them as important and presented to accident and emergency (A&E) where AF was detected. On the other hand, the majority of patients who experienced chronic or non-specific symptoms such as palpitations, dizziness, reduced exercise tolerance, or breathlessness, attributed them to old age or pre-existing conditions and therefore did not act on them:

*‘Oh I was feeling terribly dizzy. I couldn’t even stand up, even if I did stand up I fell back on the settee, and I was like that for about 2 hours. And of course we had to get the ambulance out.’* (Patient [P]011, female, aged 83 years)

*‘Yeah, I used to get out of breath a little bit. Not every time, but I thought well I’m mid 70s or whatever, I’ve got to expect it. It didn’t bother me. So I just rested and it corrected itself … it didn’t cause me any trouble and I could still carry on.’* (P035, male, aged 77 years)

Despite being aware of the onset of symptoms many did not infer illness or a need to see a healthcare professional:

*‘Why bother? I didn’t feel ill or anything like that so you’re mithering people if you do. I’m not that kind, not with people from medics. I won’t do it.’* (P055, male, aged 80 years)

Some patients took the opportunity to mention the symptoms when attending the GP for another reason:

*‘I was seeing my GP about something else … I had an attack of gout in the summer, first time ever, so I was having a chat to the GP about that and did I want medication … And I just thought oh while I’m here I’ll just mention I’ve been getting rather breathless.’* (P012, female, aged 73 years)

#### Lack of awareness

Lack of awareness of AF was a key bottleneck in the appraisal process and a driver of delay in seeking help from a healthcare professional. Most participants had not heard of AF before diagnosis and did not have knowledge of the signs or symptoms. Patients did not associate symptoms such as breathlessness and reduced fitness with the heart, and chest-related symptoms did not align with their understanding of heart conditions.

There was also a lack of awareness of the significance of an irregular pulse, for example, one patient had observed their blood pressure monitor flash ‘irregular heartbeat’ for a couple of years without taking action. Another patient was aware of having an irregular pulse as described below:

*‘I feel my pulse at certain times. I’m aware that it just doesn’t feel constant. But then maybe that’s normal, I don’t know.’* (P035, male, aged 77 years)

Lack of patient awareness also emerged in clinician interviews and a couple of GPs described how this impacts the consultation:

*‘And I think language is important too because not everybody knows what palpitations are. I’ll ask them* [patients] *if they have any palpitations and I can just tell they don’t have a clue what that means. Some of them may say “I don’t know what you mean Doc”. Some of them will just say “No” because they don’t want to be embarrassed. I think some — again, it’s important to explain to the patient what you mean by a palpitation.’* (HCP04)

#### Patients’ experiences in primary care

Interactions with primary care was a central theme in patients’ narratives. Patients described two types of interaction: instances where they had initiated a GP appointment as a result of their symptoms and instances where AF had been detected incidentally. Patients who initiated a GP appointment were motivated by concern that the symptoms could be serious because they kept recurring and/or were having an impact on their day-to-day activities:

*‘The last year I had a fit of feeling quite uncomfortable. I had quite uncomfortable, well chest discomfort really is the best way I can describe it. And then took a couple of aspirin and that went away but for the next few weeks I had some really tiresome problems* [that] *would go on for, oh 3 or 4 hours, which was unheard of for me, and I also felt really tired and breathless and that made me go to the doctor.’* (P030, female, aged 74 years)

Patients’ experiences when they presented to a GP with symptoms ranged from the GP immediately suspecting and detecting AF, to instances where GPs initially attributed symptoms to comorbid conditions. Some patients reported feeling ‘fobbed off’ or ‘dismissed’, and one patient consequently consulted a cardiologist privately.

The pathway for patients with paroxysmal AF was particularly complex; patient narratives describe ‘churn’ in the search for a diagnosis involving several visits to the GP. One patient who was being treated as having AF did not have a definitive diagnosis as their Holter test did not detect AF and they were awaiting further monitoring. Electrocardiogram (ECG) tests were done in the practice, but all patients who required Holter tests were referred to secondary care, where they would encounter wait times of up to 6 months. There were no differences in symptoms reported by male and female patients, but all the patients who reported feeling dismissed or who experienced a churn in their journey to diagnosis were female.

Patients also described incidental detection of symptomatic and asymptomatic AF during routine appointments in primary care. Incidental detection usually related to annual hypertension reviews; however, one diabetic patient’s AF was detected by a podiatrist during a routine foot examination. There were also examples of missed opportunities to detect AF in primary care. One patient reported a previous irregular pulse detected in primary care that was not investigated.

The majority of cases of AF were detected in primary care, but a few patients had their AF incidentally detected in secondary care during pre-operative assessments or during an inpatient stay. For example, two patients were diagnosed on the ward following a stroke. There were a few participants under the care of a cardiologist for pre-existing cardiac conditions such as valvular heart disease who were diagnosed incidentally following routine investigations.

#### Clinician knowledge and practice

Interviews with GPs demonstrated that GPs were knowledgeable about AF, its symptoms, and the NICE guidelines on detection and management. Generally, GPs felt they had the knowledge required to detect and manage AF, and kept up to date with changes in the NICE guidelines. Regardless, they reported not having received any formal training on the detection of AF additional to that received in medical school. GPs had contrasting views as to whether they required additional training for AF, with the majority feeling that this was neither required nor feasible. A minority felt training would be useful, and there was no consensus how this should be delivered, with some preferring videos or virtual sessions while others preferred face-to-face sessions.

GPs reported varying practices regarding opportunistic screening through pulse palpation; a few did this routinely while others did this only when they suspected AF. Consequently, the frequency of detecting AF varied, with one GP reporting they detected new AF once every 2 weeks; some GPs hardly detected AF; and one GP’s experience of AF was only through reports from secondary care or incidental findings on ECG. The nurse, on the other hand, reported routine pulse palpation as part of patients’ annual reviews, with a yield of finding a new case of AF weekly to fortnightly:

*‘I mean I’d probably hazard a guess of once every three months directly myself. And most often that would be because I’ve felt an irregular pulse, which might be a completely different — the patient might have come there with something else completely different and I’ve felt their pulse, or it may be that patients have come in with more sort of classic symptoms and obviously I’ve gone down that diagnostic route.’* (HCP04)

There were also variations in GPs’ practice in terms of referrals for a Holter test, and the presence of symptoms and/or risk factors often determined which patients were referred for ambulatory ECG monitoring, even though many of the GPs perceived AF to be largely asymptomatic:

*‘Yeah, again, I think that’s a problem, because it cannot always be permanent, can it? So if they’re symptomatic, so palpitations usually, I would refer to cardiology for a 24-hour tape or something like that, 72-hour tape, whatever. So persistent palpitations with a normal ECG I’d refer to cardio. But I suppose if the symptoms are a bit more subtle, there’s a danger that they could be reassured without further monitoring if the ECG is normal, you know, you’d kind of rule it out*.’ (HCP01)

*‘I think for that particular case, because they were older, there were risk factors and I had felt an irregular pulse, I would be doing 24-hour tape without question. I wouldn’t think about that, because I’d want to try and be assured that I wasn’t missing an AF. So those ones are pretty easy for me to decide. They’d be referred.’* (HCP04)

#### Digital technologies

Pathways to diagnosis included AF detected by digital technologies, most commonly smartwatches. The few patients who had smartwatch-detected AF were also symptomatic, and the smartwatch was the deciding factor in seeing a healthcare professional. Patients with smartwatch-detected AF had a positive response from healthcare professionals, and AF was confirmed with an ECG:

*‘I’ve had a Fitbit for a long time, and I changed to a new one because the old one died on me. And it has a heart monitor. And it started sending me messages ... I was a bit hesitant about saying “My Fitbit is telling me”. So I was a bit worried because I thought she* [GP] *might think “I did five years at medical school thank you very much” but she was lovely and she had a look at it … they also got me an ECG. And so again, that confirmed it. And so then I was in the system and they’ve now sent me a letter to say they’re offering me a cardioversion at the end of this month.’* (P033, female, aged 72 years)

These accounts of smartwatch-detected AF aligned with the experiences of primary care clinicians who reported a trend of patients presenting with smartwatch-detected AF, particularly in the more affluent areas. GPs with such encounters reported that they usually request ECGs, which usually confirm AF, although one GP was wary about taking all smartwatch-detected AF seriously. The nurse reported that patients regularly came in with smartwatch-detected AF. One patient and two GPs reported using KardiaMobiles to detect AF. The patient was advised to purchase a KardiaMobile to monitor for AF as ECG had not detected AF and there was a long wait for a Holter test in their area. Both GPs found it very useful to have the KardiaMobile at hand during consultations and used their personal devices. A third GP mentioned they were trying to convince GP partners at their practice to purchase a cardio monitor:

*‘I’m also a big fan of the cardio monitors. I use that daily, certainly weekly, because I think it’s a handy tool. And the number of patients I’ve identified with atrial fibrillation, probably in the last 6 months, has probably been about four, five patients. And that’s incidental atrial fibrillation.’* (HCP03)

One of the practices was using a novel digital technology, FibriCheck, an app that measures the heart rhythm by placing the finger on the camera of a smartphone.

#### Constraints of AF detection in primary care

GPs acknowledged that non-specific symptoms, such as palpitations and breathlessness, were associated with a range of conditions that could include AF:

*‘Yeah, I mean something like shortness of breath, because it’s such a wide range of causes, ECG isn’t normally, if it’s just shortness of breath, isn’t normally near the top of my list as a test to organise.’* (HCP07)

*‘These days we see complex comorbidity, so somebody with COPD* [chronic obstructive pulmonary disease]*, with shortness of breath that’s got worse isn’t uncommon. And in amongst that I suppose you’ve got to think “Is this just their COPD getting worse? Is there an infection?” or actually “Is there something completely new that’s happening that we don’t know about?”’* (HCP04)

GPs’ accounts alluded to variations in diagnostic capability and capacity. Some practices had an external organisation interpret their ECGs whereas some practices had to interpret them in-house, and were not always confident of their interpretation skills:

*‘I think from a practice point, having that easy access to the diagnostics, whether we need training around those particular diagnostics, particularly the ECGs. Many GPs are probably very deskilled in reading ECGs* … *Yeah. So we have a machine but we don’t have a member of staff trained.’* (HCP06)

In addition, a lack of access to Holter tests in primary care led to delays in diagnosis, leading some GPs to start anticoagulation while patients waited for their Holter tests. It was suggested having access to the Holter test in primary care would facilitate AF detection:

*‘Nowadays it can be several months* [until a patient has access to a Holter test] *which is not ideal if you’ve got somebody flipping in and out of AF. It’s not ideal at all. I have to say — I’ve got a KardiaMobile.’* (HCP08)

*‘I think we need something, Primary Care, it would be useful if we could fit more 24-hour ECGs, 72-hour ECGs. Because we can do it for blood pressure machines. So we can send patients home with a blood pressure machine attached for 24, 48 hours. But we can’t with ECG.’* (HCP05)

Interviews revealed the widespread use of remote consultations in primary care meant GPs were not able to examine patients opportunistically. In addition, there was concern that some practices were conducting patients’ annual hypertension reviews remotely using self-reported readings. Other limitations included using mechanical blood pressure devices as opposed to a manual blood pressure machine with a stethoscope, and difficulty getting GP appointments. Healthcare assistants were reported to be taking on more responsibility such as blood pressure checks, again reducing clinician opportunities to detect AF. Some primary care clinicians reported they had trained their healthcare assistants to do manual pulse palpation, but this did not appear to be standard practice. Workload pressures meant it was not always possible to do an ECG immediately, thereby missing the window of opportunity when patients are in AF.

## Discussion

### Summary

In this qualitative study of 30 patients with newly diagnosed AF and 10 primary care clinicians, patients described diverse pathways to diagnosis of AF. Our findings highlight the significance of symptoms in the detection of AF, with most patients experiencing symptoms. Two factors emerged as determinants of early presentation in patients with symptomatic AF: acuteness of onset and perceived importance. Patients experiencing the same non-specific symptoms perceived them differently, with some inferring illness and seeing a doctor and others dismissing them as not serious. A delay between symptom onset and presenting to a healthcare professional was common; not all symptomatic patients in the study took the step to see a healthcare professional and some were diagnosed incidentally. On the other hand, all patients with acute onset went to A&E where AF was diagnosed. Primary care was central to patients’ pathways to diagnosis, and patients’ narratives were corroborated by primary clinicians’ accounts of opportunistic screening and non-specificity of symptoms associated with AF. Our study also indicated a trend of smartwatch-detected AF. Interviews with primary care clinicians indicated that pressures on primary care, task shifting, and inequities in diagnostic capability and capacity limit the detection of AF.

### Strengths and weaknesses

Our study has several strengths. Identifying patients from electronic records ensured that only eligible patients were invited, and the inclusion criterion within 6 months of diagnosis minimised the risk of recall bias. Open-ended questions allowed patients to narrate their experiences in the sequence in which events occurred. In addition, we took steps to ensure the trustworthiness of the research.^15^ These include methodological triangulation by interviewing patients and healthcare professionals, investigator triangulation through double-coding a sample of the interviews and team involvement in developing themes, and considering areas of convergence and divergence in patient and clinician interviews. In terms of limitations, we only included patients who received a diagnosis, not the experiences of patients who had palpitations or other symptoms that were found to be caused by something other than AF. Our age limit of 50 years meant our study did not include pathways of younger patients with AF, which may be more convoluted as they do not fit within the age profile for AF. Also, the patient participants were predominantly of white British ethnicity despite attempts to recruit a diverse sample by including practices in areas with ethnic diversity. Our sample size is insufficient to detect rarely occurring themes.

### Comparison with the literature

Our study indicates that lack of awareness of AF is a key bottleneck in the appraisal process and a driver of delay in seeking help from a healthcare professional. Lack of awareness of AF has been reported in previous research.^16^ Two qualitative studies have previously found that patients had limited knowledge of symptoms of AF, leading to non-specific symptoms being dismissed as not important and a delay in seeking treatment.^17,18^ However, these studies focused on symptom interpretation, and our study is the first, to our knowledge, to explore the patient pathway to diagnosis according to Andersen’s model, and includes new findings regarding experiences in the health system and causes of delay within the health system. The pathways to diagnosis of symptomatic AF as described by participants are largely consistent with Andersen’s model to the point of diagnosis, and patients’ narratives indicate intervals between recognition of a symptom, deciding to seek medical care, first presentation to a healthcare professional, and subsequent processes leading to diagnosis.^12^ Nevertheless, a small group of symptomatic patients in our study did not take the step to initiate consultation with a healthcare professional and were diagnosed incidentally.

To our knowledge, our study is the first to explore primary care clinicians’ experiences in detecting AF in primary care. The limitations, such as pressures on primary care and post-COVID-19 pandemic ways of working, are consistent with contemporary literature in other (non-AF) contexts.^19,20^ The findings regarding AF detection using digital technologies are supported by literature that indicates increasing detection of AF by smartwatches and mobile ECG devices.^21–23^ Nevertheless, our study is the first, to our knowledge, to report primary care clinicians’ experiences with device-detected AF in day-to-day practice.

### Implications for practice

Within the context of the literature, our findings suggest that improved public awareness of AF and its signs and symptoms is likely to impact appraisal and help-seeking. Previous studies in cancer have shown that improved public awareness of particular cancers is associated with significant increases in detection, subsequently resulting in timely medical intervention and improved prognosis.^24,25^

It is also important that clinicians are aware of the danger of missing intermittent AF and refer patients for Holter tests. Our findings indicate that there is often a delay to performing Holter tests; GPs should be encouraged to use mobile ECG technologies such as KardiaMobile and app-based technologies like FibriCheck.

Our study suggests that the detection of AF can be improved in a number of ways. First, opportunistic screening for AF in primary care by pulse palpation is already recommended;^6,26–28^ our data reinforce this practice among GPs and nurses. Upskilling healthcare assistants and other primary care roles to opportunistically screen for AF could potentially increase the detection of AF, and evidence is needed on the feasibility and effectiveness of this approach. Second, addressing bottlenecks in the healthcare system that lead to delays in diagnosis, such as inequity in diagnostics, ECG access and interpretation, and lack of Holter tests in primary care could improve detection. In addition, remote consultations are increasingly used in primary care, reducing the opportunities to detect AF; our study suggests that annual hypertension reviews are important for the detection of AF, and there is a need to consider protecting these as face-to-face appointments. Third, there are some areas where further research and guidance is required; our study suggests that patients with AF commonly present to primary care with non-specific symptoms. The range of symptoms described by patients before diagnosis has previously been reported, but not all are listed in the NICE guidelines. The current guidelines list breathlessness, palpitations, syncope or dizziness, chest discomfort, and stroke or transient ischaemic attack,^9^ but not fatigue and reduced fitness or exercise tolerance. Also, further research on the profile of patients likely to have undiagnosed AF will facilitate detection in primary care by helping GPs identify AF.

## Data

The authors confirm that the data supporting the findings of this study are available within the article and its supplementary materials.
